# 3-[2-(3-Methyl-2-oxo-1,2-dihydro­quinoxalin-1-yl)eth­yl]oxazolidin-2-one

**DOI:** 10.1107/S1600536809028736

**Published:** 2009-07-29

**Authors:** Ahoya Anothane Caleb, Rachid Bouhfid, El Mokhtar Essassi, Lahcen El Ammari

**Affiliations:** a Laboratoire de Chimie Organique Hétérocyclique, Pôle de Compétences, Pharmacochimie, Av Ibn Battouta, BP 1014, Faculté des Sciences, Université Mohammed V-Agdal, Rabat, Morocco; b Laboratoire de Chimie du Solide Appliquée, Faculté des Sciences, Université Mohammed V-Agdal, Avenue Ibn Battouta, BP 1014, Rabat, Morocco

## Abstract

The title heterocyclic compound, C_14_H_15_N_3_O_3_, is a new synthetic mol­ecule containing oxazolidine and quinoxaline rings. It is built up from two fused six-membered rings linked to a five-membered oxazolidin-2-one ring by a C_2_ chain. Both ring systems are essentially planar [maximum deviation = 0.894 (3) Å, r.m.s. deviation = 0.0043 Å]. The structure is held together by van der Waals forces [electrostatic interactions between dipoles, O⋯C = 3.002 (2) Å] between mol­ecules and by weak π–π stacking between symmetry-related mol­ecules, with an inter­planar distance of 3.579 Å and a centroid–centroid distance of 3.800 (1) Å. Inter­molecular C—H⋯O hydrogen bonds are also observed in the crystal structure.

## Related literature

For the biological activity of 3–2(-(3-methyl-2-oxoquinoxalin-1(2*H*)-yl) eth­yl)oxazolidin-2-one, see: Ferfra (2001 ▶); Habib & El-hawash (1997 ▶); Romer *et al.* (1995 ▶). For pharmaceutical agrochemicals, see: Badran *et al.* (2003 ▶); Madhusudhan *et al.* (2004 ▶); Soad *et al.* (2006 ▶); Sriharsha & Shashikanth (2006 ▶); Sarro *et al.* (2002 ▶). For a related structure, see: Doubia *et al.* (2007 ▶).
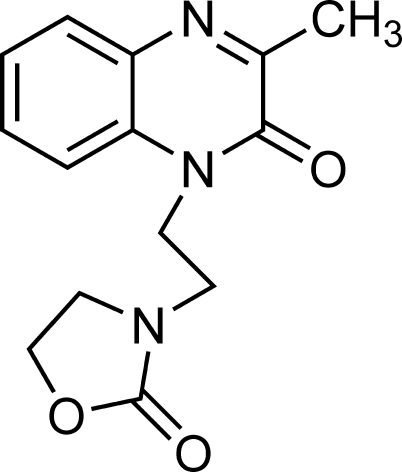


## Experimental

### 

#### Crystal data

C_14_H_15_N_3_O_3_
*M* *_r_* = 273.29
Monoclinic, 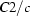

*a* = 12.280 (3) Å
*b* = 10.736 (3) Å
*c* = 20.406 (4) Å
β = 102.32 (1)°
*V* = 2628.3 (11) Å^3^
*Z* = 8
Mo *K*α radiation
μ = 0.10 mm^−1^
*T* = 298 K
0.28 × 0.17 × 0.12 mm

#### Data collection

Bruker X8 APEXII CCD area-detector diffractometer
Absorption correction: none
21237 measured reflections
4108 independent reflections
2727 reflections with *I* > 2σ(*I*)
*R*_int_ = 0.033

#### Refinement

*R*[*F* ^2^ > 2σ(*F* ^2^)] = 0.050
*wR*(*F* ^2^) = 0.157
*S* = 1.04
4108 reflections
204 parameters
H atoms treated by a mixture of independent and constrained refinement
Δρ_max_ = 0.29 e Å^−3^
Δρ_min_ = −0.24 e Å^−3^

### 

Data collection: *APEX2* (Bruker, 2005 ▶); cell refinement: *SAINT* (Bruker, 2005 ▶); data reduction: *SAINT*; program(s) used to solve structure: *SHELXS97* (Sheldrick, 2008 ▶); program(s) used to refine structure: *SHELXS97* (Sheldrick, 2008 ▶); molecular graphics: *ORTEP-3 for Windows* (Farrugia, 1997 ▶) and *PLATON* (Spek, 2009 ▶); software used to prepare material for publication: *WinGX* (Farrugia, 1999 ▶).

## Supplementary Material

Crystal structure: contains datablocks global, I. DOI: 10.1107/S1600536809028736/fj2233sup1.cif

Structure factors: contains datablocks I. DOI: 10.1107/S1600536809028736/fj2233Isup2.hkl

Additional supplementary materials:  crystallographic information; 3D view; checkCIF report

## Figures and Tables

**Table 1:** Hydrogen-bond geometry (Å, °)

*D*—H⋯*A*	*D*—H	H⋯*A*	*D*⋯*A*	*D*—H⋯*A*
C7—H5⋯O3^i^	0.98 (2)	2.54 (2)	3.462 (2)	157 (2)
C10—H10*A*⋯O3^i^	0.97	2.43	3.348 (2)	157

Symmetry code: (i) 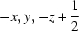.

**Fig. 1.**
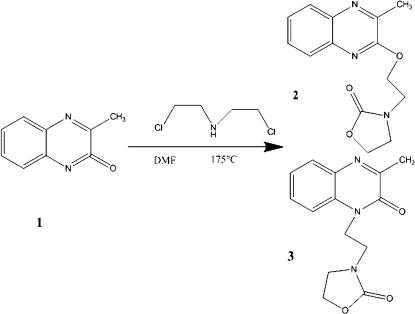
: Schematic of the chemical reaction leading to the title compound.

**Fig. 2.**
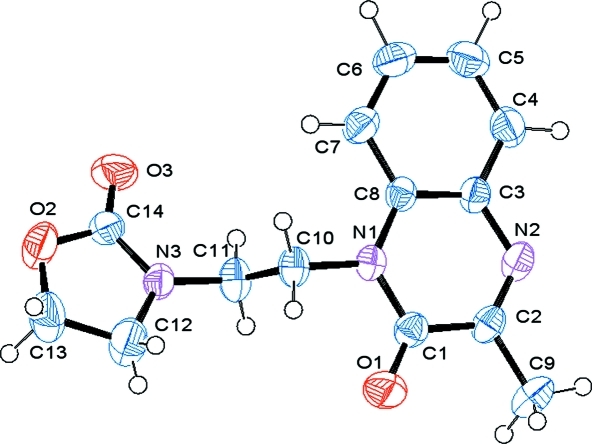
: Molecular structure of the title compound with the atom-labelling scheme. Displacement ellipsoids are drawn at the 50% probability level. H atoms are represented as small small circles.

**Fig. 3.**
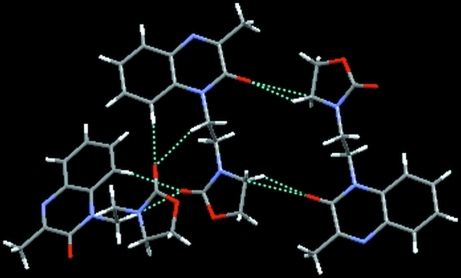
: Partial packing view showing the C—H···O interactions (dashed lines).

## supplementary crystallographic information

### Comment

The heterocyclic compounds to 5 or 6 Chains occupying a capital in fields as varied,quinoxalines pharmacy (Madhusudhan *et al.* 2004 and Sarro *et al.*2002) in agrochemicals (Romer *et al.* 1995, Habib *et al.* 1997) biology (Ferfra 2001)*etc*. The quinoxalines and the oxazolidines are subjets of numerous articles in describing the synthesis of new derivatives presentery antibacterial properties (Badran *et al.* 2003, Sriharsha *et al.* 2006) and anti tumor (Soad *et al.* 2006). We describe here the synthesis of compound 3 to side of the compound 2 per share on the dichlorodiéthylmine quinoxaline-2-one fusion as show in the chemical structural diagram (Fig.1).

The 3–2(-(3-methyl-2-oxoquinoxalin-1(2*H*)-yl)ethyl)oxazolidin-2-one (I) molecule structure is built up from two fused six-membered rings linked to a five-membered ring (oxazolidin-2-one) by an ethylic groupe. The both rings are essentially planar and forms a dihedral angle of 20.46 (6)° with the oxazolidin-2-one ring. The molecular structure of (I) is shown in Fig.2. The geometric parameters (bond lenghths and angles) are very similar to those observed in previously reported structures (Doubia *et al.* 2007).

An intermolecular C—H···O hydrogen bond is observed in the cristal structure as shown in the partial plot of the structure (Fig.3). Furthermore, the structure is stabilized by Van der Waals forces and together by weak slipped π-π stzcking between symmetry related molecules (C to C ring) with interplanar distance of 3.579 Å and centroid to centroid vector of 3.800 (1) Å.

### Experimental

It reacted 0.0125 moles of quinoxaline-2-one with 2.66 moles of dichlorodiéthylamine in 40 ml dimethyl formamide in the presence of 2.87 moles of K_2_CO_3_ and a few milligrams of BTBA. The mixture was brought to reflux in a bath of sand magnetic stirring for 6 h. After vacuum concentration, the separation of compounds was done by column chromatography eluant 4 / 6(hexane - ethyl acetate). Recrystallization occurred in the same eluent. This compound was obtained in 60% and his melting point is 175°C.

### Refinement

All H atoms were located in a difference map and refined without any distance restraints.

### Crystal data

**Table:**

C_14_H_15_N_3_O_3_	*F*(000) = 1152
*M**_r_* = 273.29	*D*_x_ = 1.381 Mg m^−^^3^
Monoclinic, *C*2/*c*	Melting point: 448 K
Hall symbol: -C 2yc	Mo *K*α radiation, λ = 0.71073 Å
*a* = 12.280 (3) Å	Cell parameters from 21279 reflections
*b* = 10.736 (3) Å	θ = 2.6–30.9°
*c* = 20.406 (4) Å	µ = 0.10 mm^−^^1^
β = 102.32 (1)°	*T* = 298 K
*V* = 2628.3 (11) Å^3^	Prism, colourless
*Z* = 8	0.28 × 0.17 × 0.12 mm

### Data collection

**Table:**

Bruker X8 APEXII CCD area-detector diffractometer	*R*_int_ = 0.033
graphite	θ_max_ = 30.9°, θ_min_ = 2.6°
φ and ω scans	*h* = −17→17
21237 measured reflections	*k* = −15→15
4108 independent reflections	*l* = −26→29
2727 reflections with *I* > 2σ(*I*)	

### Refinement

**Table:**

Refinement on *F*^2^	Primary atom site location: structure-invariant direct methods
Least-squares matrix: full	Secondary atom site location: difference Fourier map
*R*[*F*^2^ > 2σ(*F*^2^)] = 0.050	Hydrogen site location: inferred from neighbouring sites
*wR*(*F*^2^) = 0.157	H atoms treated by a mixture of independent and constrained refinement
*S* = 1.04	*w* = 1/[σ^2^(*F*_o_^2^) + (0.081*P*)^2^ + 0.6684*P*] where *P* = (*F*_o_^2^ + 2*F*_c_^2^)/3
4108 reflections	(Δ/σ)_max_ = 0.001
204 parameters	Δρ_max_ = 0.29 e Å^−^^3^
0 restraints	Δρ_min_ = −0.24 e Å^−^^3^

### Special details

**Table:**

Geometry. All e.s.d.'s (except the e.s.d. in the dihedral angle between two l.s. planes) are estimated using the full covariance matrix. The cell e.s.d.'s are taken into account individually in the estimation of e.s.d.'s in distances, angles and torsion angles; correlations between e.s.d.'s in cell parameters are only used when they are defined by crystal symmetry. An approximate (isotropic) treatment of cell e.s.d.'s is used for estimating e.s.d.'s involving l.s. planes.
Refinement. Refinement of *F*^2^ against ALL reflections. The weighted *R*-factor *wR* and goodness of fit *S* are based on *F*^2^, conventional *R*-factors *R* are based on *F*, with *F* set to zero for negative *F*^2^. The threshold expression of *F*^2^ > σ(*F*^2^) is used only for calculating *R*-factors(gt) *etc*. and is not relevant to the choice of reflections for refinement. *R*-factors based on *F*^2^ are statistically about twice as large as those based on *F*, and *R*- factors based on ALL data will be even larger.

### Fractional atomic coordinates and isotropic or equivalent isotropic displacement parameters (Å^2^)

**Table:**

	*x*	*y*	*z*	*U*_iso_*/*U*_eq_	
O1	0.11176 (11)	0.25753 (10)	0.54216 (6)	0.0630 (3)	
O2	−0.13309 (10)	0.52714 (11)	0.26113 (5)	0.0598 (3)	
O3	−0.13793 (10)	0.32026 (12)	0.24503 (6)	0.0662 (4)	
N1	0.11384 (8)	0.15986 (10)	0.44405 (5)	0.0353 (2)	
N2	0.16192 (10)	−0.06171 (10)	0.51654 (5)	0.0422 (3)	
N3	−0.06491 (10)	0.40729 (10)	0.34753 (5)	0.0411 (3)	
C1	0.12314 (11)	0.16145 (12)	0.51219 (6)	0.0387 (3)	
C2	0.14895 (11)	0.04062 (12)	0.54680 (6)	0.0385 (3)	
C3	0.15128 (10)	−0.05948 (12)	0.44748 (6)	0.0375 (3)	
C4	0.16612 (13)	−0.17122 (14)	0.41519 (8)	0.0499 (4)	
H4	0.1830 (15)	−0.2426 (16)	0.4433 (9)	0.064 (5)*	
C5	0.15685 (13)	−0.17446 (16)	0.34713 (8)	0.0550 (4)	
H5	0.0990 (14)	0.1222 (17)	0.3136 (8)	0.061 (5)*	
C6	0.13209 (13)	−0.06520 (17)	0.31028 (8)	0.0524 (4)	
H6	0.1300 (16)	−0.0693 (17)	0.2637 (11)	0.070 (6)*	
C7	0.11673 (12)	0.04585 (15)	0.34045 (7)	0.0452 (3)	
H7	0.1688 (16)	−0.2533 (17)	0.3259 (9)	0.064 (5)*	
C8	0.12719 (10)	0.05051 (11)	0.40996 (6)	0.0341 (3)	
C9	0.15977 (14)	0.04223 (15)	0.62100 (7)	0.0522 (4)	
H9A	0.1775	−0.0400	0.6386	0.090*	
H9B	0.0907	0.0690	0.6313	0.080*	
H9C	0.2181	0.0987	0.6409	0.071 (6)*	
C10	0.08780 (11)	0.27856 (12)	0.40844 (7)	0.0414 (3)	
H10A	0.1230	0.2811	0.3702	0.053 (4)*	
H10B	0.1171	0.3469	0.4381	0.047 (4)*	
C11	−0.03761 (11)	0.29325 (13)	0.38470 (7)	0.0457 (3)	
H11A	−0.0670	0.2231	0.3565	0.062 (5)*	
H11B	−0.0723	0.2934	0.4232	0.069 (5)*	
C12	−0.0550 (2)	0.52845 (15)	0.37655 (9)	0.0767 (6)	
H12A	0.0221	0.5485	0.3962	0.090*	
H12B	−0.0990	0.5361	0.4106	0.088*	
C13	−0.10029 (16)	0.61022 (15)	0.31685 (9)	0.0625 (5)	
H13A	−0.1637	0.6578	0.3242	0.081 (6)*	
H13B	−0.0435	0.6676	0.3088	0.089 (7)*	
C14	−0.11293 (11)	0.40817 (14)	0.28215 (7)	0.0429 (3)	

### Atomic displacement parameters (Å^2^)

**Table:**

	*U*^11^	*U*^22^	*U*^33^	*U*^12^	*U*^13^	*U*^23^
O1	0.0931 (9)	0.0442 (6)	0.0505 (6)	0.0055 (6)	0.0130 (6)	−0.0100 (5)
O2	0.0708 (8)	0.0578 (7)	0.0454 (6)	0.0030 (5)	0.0000 (5)	0.0146 (5)
O3	0.0708 (8)	0.0718 (8)	0.0502 (6)	0.0112 (6)	0.0005 (5)	−0.0246 (6)
N1	0.0362 (5)	0.0336 (5)	0.0352 (5)	0.0005 (4)	0.0053 (4)	0.0056 (4)
N2	0.0472 (6)	0.0398 (6)	0.0353 (6)	−0.0030 (5)	−0.0010 (4)	0.0057 (4)
N3	0.0502 (7)	0.0332 (5)	0.0361 (6)	0.0048 (4)	0.0009 (5)	0.0017 (4)
C1	0.0397 (6)	0.0385 (6)	0.0363 (6)	−0.0027 (5)	0.0048 (5)	−0.0001 (5)
C2	0.0369 (6)	0.0425 (7)	0.0329 (6)	−0.0078 (5)	0.0005 (5)	0.0033 (5)
C3	0.0360 (6)	0.0370 (6)	0.0368 (6)	0.0008 (5)	0.0016 (5)	0.0028 (5)
C4	0.0525 (8)	0.0400 (7)	0.0528 (8)	0.0061 (6)	0.0013 (6)	−0.0023 (6)
C5	0.0513 (9)	0.0561 (9)	0.0557 (9)	0.0062 (7)	0.0075 (7)	−0.0160 (7)
C6	0.0475 (8)	0.0734 (11)	0.0373 (7)	0.0010 (7)	0.0110 (6)	−0.0072 (7)
C7	0.0434 (7)	0.0566 (8)	0.0360 (7)	0.0010 (6)	0.0091 (5)	0.0063 (6)
C8	0.0289 (6)	0.0381 (6)	0.0345 (6)	0.0004 (4)	0.0050 (4)	0.0036 (5)
C9	0.0595 (9)	0.0622 (9)	0.0319 (6)	−0.0168 (7)	0.0030 (6)	0.0035 (6)
C10	0.0425 (7)	0.0341 (6)	0.0463 (7)	−0.0008 (5)	0.0066 (5)	0.0103 (5)
C11	0.0426 (7)	0.0387 (7)	0.0533 (8)	0.0001 (5)	0.0044 (6)	0.0121 (6)
C12	0.1231 (18)	0.0380 (8)	0.0533 (10)	0.0076 (9)	−0.0162 (10)	−0.0075 (7)
C13	0.0684 (11)	0.0380 (8)	0.0734 (11)	0.0008 (7)	−0.0019 (8)	0.0086 (7)
C14	0.0399 (7)	0.0513 (8)	0.0368 (6)	0.0074 (6)	0.0067 (5)	−0.0016 (6)

### Geometric parameters (Å, °)

**Table:**

O1—C1	1.2223 (17)	C5—H7	0.976 (19)
O2—C14	1.3532 (18)	C6—C7	1.373 (2)
O2—C13	1.434 (2)	C6—H6	0.95 (2)
O3—C14	1.2080 (18)	C7—C8	1.3975 (18)
N1—C1	1.3708 (16)	C7—H5	0.984 (18)
N1—C8	1.3921 (16)	C9—H9A	0.9600
N1—C10	1.4680 (16)	C9—H9B	0.9600
N2—C2	1.2868 (18)	C9—H9C	0.9600
N2—C3	1.3874 (17)	C10—C11	1.5208 (19)
N3—C14	1.3387 (17)	C10—H10A	0.9700
N3—C12	1.4237 (19)	C10—H10B	0.9700
N3—C11	1.4415 (16)	C11—H11A	0.9700
C1—C2	1.4787 (18)	C11—H11B	0.9700
C2—C9	1.4915 (19)	C12—C13	1.508 (2)
C3—C4	1.3991 (19)	C12—H12A	0.9700
C3—C8	1.4041 (17)	C12—H12B	0.9700
C4—C5	1.369 (2)	C13—H13A	0.9700
C4—H4	0.953 (17)	C13—H13B	0.9700
C5—C6	1.392 (2)		
			
C14—O2—C13	109.55 (11)	C2—C9—H9B	109.5
C1—N1—C8	121.61 (10)	H9A—C9—H9B	109.5
C1—N1—C10	116.97 (11)	C2—C9—H9C	109.5
C8—N1—C10	121.42 (10)	H9A—C9—H9C	109.5
C2—N2—C3	118.58 (11)	H9B—C9—H9C	109.5
C14—N3—C12	112.86 (11)	N1—C10—C11	110.28 (10)
C14—N3—C11	122.27 (11)	N1—C10—H10A	109.6
C12—N3—C11	124.57 (12)	C11—C10—H10A	109.6
O1—C1—N1	121.63 (12)	N1—C10—H10B	109.6
O1—C1—C2	122.48 (12)	C11—C10—H10B	109.6
N1—C1—C2	115.89 (11)	H10A—C10—H10B	108.1
N2—C2—C1	123.75 (11)	N3—C11—C10	111.20 (11)
N2—C2—C9	120.25 (12)	N3—C11—H11A	109.4
C1—C2—C9	116.00 (12)	C10—C11—H11A	109.4
N2—C3—C4	118.03 (12)	N3—C11—H11B	109.4
N2—C3—C8	122.11 (12)	C10—C11—H11B	109.4
C4—C3—C8	119.85 (12)	H11A—C11—H11B	108.0
C5—C4—C3	120.55 (14)	N3—C12—C13	102.23 (13)
C5—C4—H4	123.4 (11)	N3—C12—H12A	111.3
C3—C4—H4	116.1 (11)	C13—C12—H12A	111.3
C4—C5—C6	119.21 (14)	N3—C12—H12B	111.3
C4—C5—H7	119.0 (11)	C13—C12—H12B	111.3
C6—C5—H7	121.8 (11)	H12A—C12—H12B	109.2
C7—C6—C5	121.64 (14)	O2—C13—C12	105.73 (13)
C7—C6—H6	120.9 (11)	O2—C13—H13A	110.6
C5—C6—H6	117.4 (11)	C12—C13—H13A	110.6
C6—C7—C8	119.63 (14)	O2—C13—H13B	110.6
C6—C7—H5	120.6 (10)	C12—C13—H13B	110.6
C8—C7—H5	119.8 (10)	H13A—C13—H13B	108.7
N1—C8—C7	122.83 (12)	O3—C14—N3	128.19 (14)
N1—C8—C3	118.06 (11)	O3—C14—O2	122.27 (13)
C7—C8—C3	119.10 (12)	N3—C14—O2	109.53 (12)
C2—C9—H9A	109.5		

### Hydrogen-bond geometry (Å, °)

**Table:**

*D*—H···*A*	*D*—H	H···*A*	*D*···*A*	*D*—H···*A*
C7—H5···O3^i^	0.98 (2)	2.54 (2)	3.462 (2)	157 (2)
C10—H10A···O3^i^	0.97	2.43	3.348 (2)	157

Symmetry codes: (i) −*x*, *y*, −*z*+1/2.
